# Stromal myofibroblasts in oral leukoplakia and oral squamous cell carcinoma

**DOI:** 10.4317/medoral.17834

**Published:** 2012-02-09

**Authors:** Eliene M. de-Assis, Luiz G.G.S. Pimenta, Edson Costa-e-Silva, Paulo E.A. Souza, Martinho C.R. Horta

**Affiliations:** 1 Department of Dentistry, Pontifícia Universidade Católica de Minas Gerais, Belo Horizonte, MG, Brazil

## Abstract

Objectives: Oral leukoplakia (OL) is the main potentially malignant disorder and oral squamous cell carcinoma (OSCC) is the most common malignancy of the oral mucosa. Stromal myofibroblasts play an important role in tumor invasion and metastasis, due to its ability to modify the extracellular matrix. This study aimed to evaluate the presence of stromal myofibroblasts in OL and OSCC. Differences in the presence of myofibroblasts among OL with distinct grades of epithelial dysplasia as well as between histologically high- and low-invasive OSCC were also assessed. Study Design: A total of 30 OL and 41 OSCC from archival formalin-fixed, paraffin-embedded specimens were evaluated. 10 samples of normal oral mucosa were used as a control. Myofibroblasts were identified by immunohistochemical detection of alpha smooth muscle actin and its presence was classified as negative, scanty or abundant. Differences in the presence of myofibroblasts among OL with distinct grades of epithelial dysplasia as well as between high- and low-invasive OSCC were analyzed using the Mann-Whitney test. Results: Myofibroblasts were not detected in normal oral mucosa and OL, whatever its histological grade. In OSCC, the presence of stromal myofibroblasts was classified as negative in 11 (26.8%), scanty in 15 (36.6%), and abundant in 15 samples (36.6%). The presence of stromal myofibroblasts was statistically higher in high-invasive OSCC than in low-invasive OSCC (p<0.05). Conclusions: Stromal myofibroblasts were not detected in OL, indicating that these cells are not important during oral carcinogenesis. Nevertheless, stromal myofibroblasts were heterogeneously detected in OSCC and its presence was higher in tumors with a more diffuse histological pattern of invasion. These findings suggest that myofibroblasts are associated with the creation of a permissive environment for tumor invasion in OSCC.

** Key words:**Leukoplakia, oral squamous cell carcinoma, myofibroblast.

## Introduction

Leukoplakia is the most common potentially malignant disorder of the oral mucosa, recently redefined as a white plaque of questionable risk having excluded other known diseases or disorders that carry no increased risk for cancer (1). Oral leukoplakia (OL) mainly affects men over 40 years old and tobacco use is its most important predisposing factor (2). OL is histologically categorized in rising grades of epithelial dysplasia and its rate of malignant transformation ranges among 0.13% to 2.2% per year (2).

Oral squamous cell carcinoma (OSCC) is one of the most common malignances worldwide (3). OSCC mainly affects men over 40 years old (4) and the most relevant risk factors for its development are tobacco and alcohol (5). The tumor may arise in any site of the oral cavity chiefly in lower lip, tongue, floor of mouth, soft palate, and gingival/alveolar ridge (4).

Myofibroblasts are differentiated fibroblasts that express alpha smooth muscle actin and have intermediate characteristics between classic fibroblasts and smooth muscle cells (6-8). Its most referred origin is the fibroblast transdifferentiation stimulated by cytokines as TGF-β1 (6). Due to its ability to modify the extracellular matrix, myofibroblasts play an important role in tumor invasion and metastasis (9,10). The presence of stromal myofibroblasts has been associated with a worse prognosis in epithelial malignant tumors (11,12) including OSCC (13,14).

The aim of this study was to evaluate the presence of stromal myofibroblasts in OL and OSCC. Differences in the presence of stromal myofibroblasts among OL with distinct grades of epithelial dysplasia as well as between histologically high- and low-invasive OSCC were also accessed.

## Material and Methods

Tissue samples

This study was approved by the local ethics committee.

A total of 30 OL and 41 OSCC from archival formalin-fixed, paraffin-embedded specimens were evaluated. 10 samples of normal oral mucosa were used as a control.

Of the 30 patients whose OL were evaluated, 17 were men and 13 were women, ranging from 33 to 81 years of age (mean age, 52 years). In OSCC samples, 30 patients were men and 11 were women, ranging from 27 to 81 years of age (mean age, 59 years).

Histological grading of OL

The histological grading of OL was performed on hematoxylin and eosin stained sections, as described elsewhere (15).

Of the 30 OL samples, 13 were classified as OL showing no or mild epithelial dysplasia and 17 were classified as OL showing moderate or severe epithelial dysplasia.

Evaluation of the pattern of invasion of OSCC

The pattern of invasion of OSSC samples was performed on hematoxylin and eosin stained sections, according to Bryne et al. (16). The pattern of invasion was classified as: grade 1 - pushing, well delineated infiltrating borders; grade 2 - infiltrating, solid cords, bands and/or strands; grade 3 - small groups or cords of infiltrating cells (n >15); grade 4 - marked and widespread cellular dissociation in small groups and/or in single cells (n <15).

Of the 41 OSCC samples, 16 were classified as low-invasive OSCC (pattern of invasion grade 1 or 2) and 25 were classified as high-invasive OSCC (pattern of invasion grade 3 or 4).

Immunohistochemistry

Myofibroblasts were identified by immunohistochemical detection of alpha smooth muscle actin. Four µm sections from the paraffin-embedded samples were used. Tissue sections were dewaxed with xylene, hydrated using graded alcohols, and treated with 0.6% H2O2 to eliminate endogenous peroxidase activity. Antigen retrieval was conducted by heating in a 0.01 M citrate buffer (pH 6.0) for 30 minutes. Subsequently, the anti - alpha smooth muscle actin monoclonal antibody was used (clone 1A4, diluted 1:100; Dako Corporation, Carpinteria, USA). The LSAB+ kit (Dako Corporation, Carpinteria, USA) was used for application of the biotinylated link antibody and peroxidase-labeled streptavidin, according to the manufacturer’s instructions. The reactive products were visualized by immersing the sections for 3 min in 0.03% diaminobenzidine solution, containing 2 mM H2O2. The sections were then counterstained with Mayer’s hematoxylin, dehydrated, and mounted. Normal vessel’s smooth muscle immunoreactivity was used as internal positive control. Negative control was determined by omission of the primary antibody.

Scoring of immunostaining results

A light microscopy was used to evaluate the immunohistochemical reactions. Alpha smooth muscle actin positive stromal cells, showing cytoplasmatic immunostaining, were considered as myofibroblasts. The presence of stromal myofibroblasts was assessed independently by two authors (HMA and MCRH) and qualitatively classified as negative, scanty or abundant, as described elsewhere (13). Doubtful cases were reanalyzed and a consensus score agreed. Samples in which no stromal myofibroblasts were detected were classified as negative. Samples showing sporadic stromal myofibroblasts were classified as scanty. Samples showing numerous and densely arranged stromal myofibroblasts were classified as abundant.

Statistical analysis

The data were analyzed by means of BioEstat 5.0 software (Optical Digital Technology, Belém, Brazil). Differences in the presence of myofibroblasts among OL with distinct grades of epithelial dysplasia as well as between high- and low-invasive OSCC were analyzed using the Mann-Whitney test. The level of significance was established at 5%.

## Results

Stromal myofibroblasts were not observed in normal oral mucosa used as a control ( Table 1).

**Table 1:**
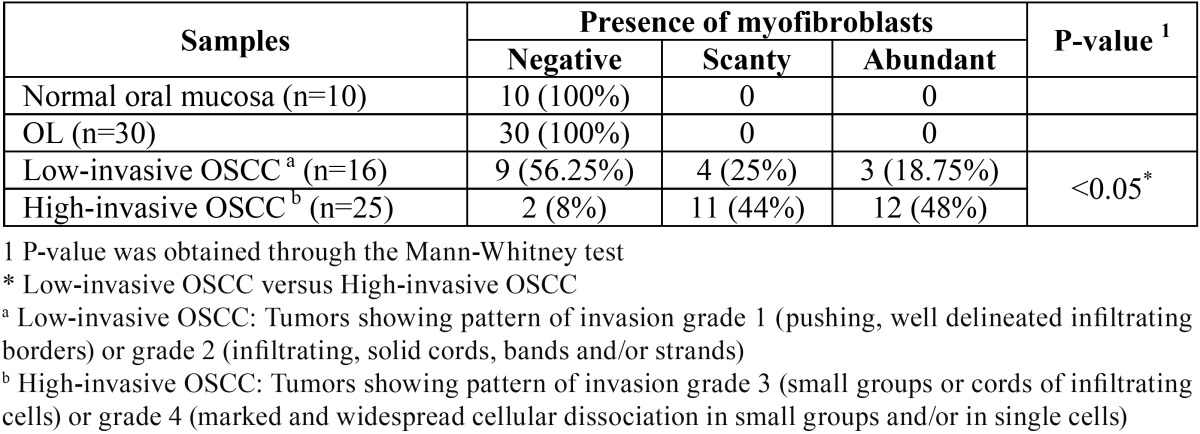
Presence of stromal myofibroblasts in normal oral mucosa, oral leukoplakia (OL) and oral squamous cell carcinoma (OSCC).

**Figure 1:**
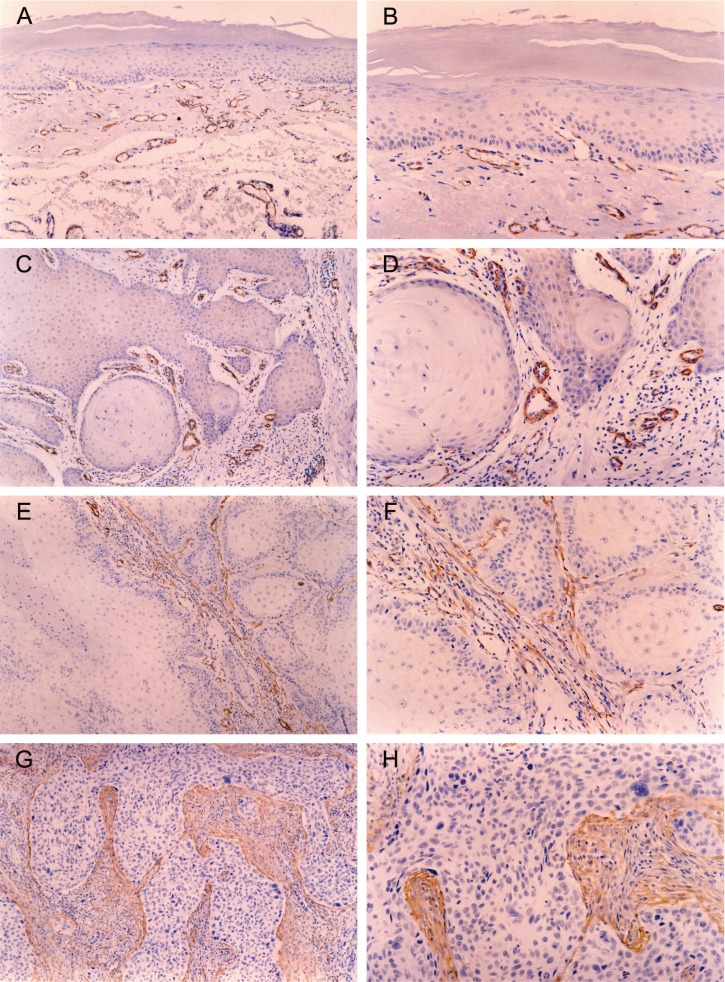
Immunohistochemical reactivity for alpha smooth muscle actin in oral leukoplakia (OL) and oral squamous cell carcinoma (OSCC). Alpha smooth muscle actin positive stromal cells, showing cytoplasmatic immunostaining, were considered as myofibroblasts. Normal vessel’s smooth muscle immunoreactivity was used as internal positive control. Myofibroblasts were not observed in OL [original magnification: x100(A) and x200(B)]. In OSCC, the presence of myofibroblasts was negative [original magnification: x100(C) and x200(D)], scanty [original magnification: x100(E) and x200(F)] or abundant [original magnification: x100(G) and x200(H)].

Stromal myofibroblasts were also not detected in any of the 30 OL samples, whatever its histological grade (Fig. 1) ( Table 1).

In the 41 samples of OSCC, the presence of stromal myofibroblasts was classified as negative in 11 (26.8%) (Fig. 1), scanty in 15 (36.6%) (Fig. 1) and abundant in 15 (36.6%) (Fig. 1).

Once OSCC samples were separated in low-invasive and high-invasive tumors, a remarkable difference in the presence of stromal myofibroblasts was observed ( Table 1). In 16 samples of low-invasive OSCC, the presence of myofibroblasts was classified as negative in 9 (56.25%), scanty in 4 (25%) and abundant in 3 (18.75%). In 25 samples of high-invasive OSCC, the presence of myofibroblasts was classified as negative in 2 (8%), scanty in 11 (44%) and abundant in 12 (48%). Consequently, the presence of stromal myofibroblasts was statistically higher in high-invasive OSCC than in low-invasive OSCC (p<0.05).

## Discussion

The term myofibroblast was introduced in the early 1970s to name fibroblasts that, under certain conditions as granulation tissue contraction, are capable of modulating toward a cell type structurally and functionally close to smooth muscle (6). A comprehensive recent definition of the myofibroblast includes light microscopy features (spindle-cell or stellate-cell morphology, palely eosinophilic cytoplasm, and an abundant pericellular matrix), immunophenotype characteristics (positivity for markers as alpha smooth muscle actin), and ultrastructure features (prominent rough endoplasmatic reticulum, a Golgi apparatus producing collagen secretion granules, peripheral myofilaments, fibronexus junctions and gap junctions) (8). Myofibroblasts synthesize and secrete cytokines, growth factors, inflammatory mediators, extracellular matrix proteins, metalloproteinases, and tissue inhibitors of metalloproteinases. Therefore, play an important role during embryogenesis, organogenesis, inflammation, would healing and cancer (6-10).

Due to its ability to modify the extracellular matrix, myofibroblasts actively participate in tumor invasion and metastasis (9,10). Metastasis is a complex process that depends on many interactions among tumor cells and the microenvironment, involving a sequence of events characterized by tumor growth and angiogenesis, detachment between the tumor cells, invasion of the extra-cellular matrix (ECM), vascular dissemination, extravasation in target organs, and establishment of secondary tumor. During the ECM invasion, the tumor cell must adhere to its components, promote its degradation by metalloproteinases, and then move through the degraded ECM (17,18). This dynamic process of ECM remodeling, called stromagenesis, is orchestrated by stromal myofibroblasts and creates a permissive environment for tumor growth, invasion and metastasis (10).

Myofibroblasts were not observed in any of the 30 OL samples evaluated, no matter what the OL histological grade ( Table 1). Similar results from prior literature have been observed as regards OL (13,19) and dysplastic epithelium in rat tongue carcinogenesis model (20). These results suggest that myofibroblasts are not present in the stroma during oral carcinogenesis, even in its advanced stages. These results also reinforce the hypothesis that, in this process, the appearance of myofibroblasts is entirely dependent on the OSCC development (20) and contact among OSCC cells and the stroma is needed to induce myofibroblast transdifferentiation (13). Nevertheless, myofibroblasts were in fact associated with the increasing grade of epithelial dysplasia in other anatomic sites, as the uterine cervix (21).

Our results showed the presence of myofibroblasts (scanty or abundant) in 73.2% of the OSCC samples. These findings are in accordance with previous reports evaluating OSCC (13,14,22-25). These results suggest that stromal remodeling induced by OSCC is characterized by gain of myofibroblasts, as originally reported by Barth et al. (22) and Lewis et al. (23). The myofibro-blast is a key cell in the stromatogenesis, a dynamic process in ECM remodeling, induced by tumor cells, that creates a permissive environment for tumor growth, local invasion and metastasis (10). Kellermann et al. (13) demonstrated that OSCC showing abundant presence of myofibroblasts are associated to higher proliferative activity of tumor cells, regional metastasis and survival, in relation to OSCC with negative and scanty myofibroblasts. Moreover, Marsh et al. (14) have recently demonstrated that the presence of stromal myofibroblasts is an effective predictor of OSCC mortality and is associated to a group of patients with aggressive tumors, regardless of tumor stage.

The presence of myofibroblasts was negative in 26.8%, scanty in 36.6%, and abundant in 36.6% of the OSCC samples. This heterogeneous presence of stromal myofibroblasts in OSCC has been previous reported (13,14,22-25). Since TGF-β1 secretion by OSCC cell lines induce oral fibroblast transdifferentiation into myofibroblasts (23,26), this heterogeneity should be explained by disparity in TGF-β1 expression among different OSCC samples (27). The role of TGF-β1 in neoplasia is complex, switching from tumor supressor in the premalignant phases of carcinogenesis to prooncogene at later stages of cancer, enhancing invasion and metastasis through epigenetic mechanisms (28).

Once OSCC samples were separated in low-invasive and high-invasive tumors, a remarkable difference in the presence of stromal myofibroblasts was observed ( Table 1). The presence of stromal myofibroblasts was statistically higher in high-invasive OSCC than in low-invasive OSCC (p<0.05). The pattern of invasion is an important histological parameter that reflects the invasive features of the tumor (16) and OSCC with more diffuse pattern of invasion presents a worse prognosis (29). Furthermore, it has been reported that OSCC that invaded in small groups or with widespread cellular dissociation (high-invasive tumors; pattern of invasion grades 3 or 4) showed a higher tendency to metastasize to regional lymph nodes when compared with OSCC that invaded in well delineated borders or in solid cords, bands and/or strands (low-invasive tumors; pattern of invasion grades 1 or 2) (30). Therefore, our findings reinforce the statement that the presence of myofibroblasts in the stroma of OSCC creates a permissive environment for tumor invasion and metastasis.

In conclusion, stromal myofibroblasts were not detected in OL, indicating that these cells are not significant during oral carcino-genesis. Nevertheless, stromal myofibroblasts were heterogeneously detected in OSCC and its presence was higher in tumors with a more diffuse histological pattern of invasion. These findings suggest that myofibroblasts are associated with the creation of a permissive environment for tumor invasion in OSCC and should play an active role in OSCC invasion and metastasis.
